# Differential Response of Salivary MMP-8 and MMP-9 to Full-Mouth Disinfection: Implications for Biomarker Sensitivity in Periodontal Therapy

**DOI:** 10.3390/dj14050283

**Published:** 2026-05-09

**Authors:** Bogdan-Constantin Vasiliu, Ionuț Tărăboanță, Alexandra Cornelia Teodorescu, Alexandru Lodbă, Gabriel Rotundu, Ecaterina Anisie, Ionut Luchian, Sorina Mihaela Solomon

**Affiliations:** 1 Grigore T. Popa University of Medicine and Pharmacy, 16 Universitatii Str., 700115 Iasi, Romania; bogdan.vasiliu@umfiasi.ro (B.-C.V.); cornelia.teodorescu@umfiasi.ro (A.C.T.); lodba.alexandru@d.umfiasi.ro (A.L.); gabriel.rotundu@umfiasi.ro (G.R.); ionut.luchian@umfiasi.ro (I.L.); sorina.solomon@umfiasi.ro (S.M.S.); 2 Department of Microbiology/Immunology, Clinical Emergency Hospital “Sfantul Spiridon”, 700106 Iasi, Romania; ecaterina.anisie@spitalspiridon.ro

**Keywords:** periodontitis, matrix metalloproteinase 8, matrix metalloproteinase 9, saliva, biomarkers, periodontal therapy, depression

## Abstract

**Background/Objectives**: Matrix metalloproteinases (MMP-8 and MMP-9) are key mediators of periodontal tissue degradation and have been proposed as salivary biomarkers of disease activity. However, their comparative responsiveness to non-surgical periodontal therapy remains insufficiently clarified. This study aimed to assess short-term changes in salivary MMP-8 and MMP-9 following full-mouth disinfection (FMD), to evaluate their association with clinical periodontal parameters, and to explore potential differences according to depressive comorbidity. **Methods**: Eighty patients were included and divided into two groups: 40 patients without depressive disorder (PAR group) and 40 patients with depressive disorder (DEP group). Clinical periodontal parameters (probing depth and clinical attachment loss) and salivary levels of MMP-8 and MMP-9 were assessed at baseline and 12 weeks following FMD. Salivary MMP-8 and MMP-9 levels were determined using the enzyme-linked immunosorbent assay (ELISA). **Results**: Significant reductions in probing depth and clinical attachment loss were observed following therapy (*p* < 0.01). Salivary MMP-9 levels decreased significantly in both groups (PAR: 2.4 to 1.1 ng/mL, *p* = 0.002; DEP: 2.6 to 1.5 ng/mL, *p* = 0.004), whereas MMP-8 did not show statistically significant changes (*p* > 0.05). Moderate positive correlations were identified between MMP-9 and clinical parameters (r = 0.46–0.51), while MMP-8 showed no significant associations. No significant differences in treatment response or biomarker dynamics were observed between groups. **Conclusions**: The findings indicate a differential salivary biomarker response to periodontal therapy, with MMP-9 showing greater responsiveness than MMP-8 under the conditions of this study. These results suggest a potential role for salivary MMP-9 in reflecting short-term periodontal changes; however, further longitudinal and controlled studies are required to establish its clinical applicability. Depressive comorbidity did not appear to influence short-term outcomes, although this finding should be interpreted cautiously due to the lack of a standardized severity assessment.

## 1. Introduction

Periodontitis is a chronic inflammatory disease characterized by the progressive destruction of the tooth-supporting structures, resulting from a complex interaction between subgingival microbiota and the host immune response [1]. Clinical parameters such as probing depth (PD) and clinical attachment loss (CAL) are routinely used to assess disease severity and treatment outcomes [2].

In addition to microbial factors, systemic and psychosocial conditions, including depressive disorders, have been increasingly recognized as potential modulators of periodontal disease. These conditions may influence both behavioral factors, such as oral hygiene practices, and biological pathways through dysregulation of the stress–inflammation axis [3,4].

Biomarkers play a crucial role in the early detection, monitoring, and prognosis of inflammatory diseases, including periodontitis [5]. In the context of periodontal inflammation, salivary biomarkers offer a non-invasive approach for assessing disease activity and therapeutic response [6].

Matrix metalloproteinases (MMPs), particularly MMP-8 and MMP-9, play a central role in periodontal tissue breakdown by mediating extracellular matrix degradation. Their presence in saliva and gingival crevicular fluid has been associated with periodontal inflammation and disease severity, suggesting their potential utility as non-invasive biomarkers [5].

Despite extensive research on MMPs in periodontal disease, their comparative responsiveness to non-surgical therapy remains insufficiently clarified, particularly when assessed in whole saliva [7]. Although both MMP-8 and MMP-9 are involved in tissue degradation, their biological behavior and diagnostic performance may differ depending on the sampling medium and temporal dynamics of disease resolution [8,9].

A key unresolved question is whether these biomarkers exhibit similar sensitivity to short-term therapeutic interventions or whether specific MMPs more accurately reflect early periodontal healing. Addressing this distinction is essential for improving the clinical utility of salivary biomarkers in monitoring periodontal treatment response [10].

Full-mouth disinfection (FMD) is a non-surgical periodontal therapy aimed at the rapid reduction of the subgingival microbial load, thereby limiting intraoral reinfection and promoting clinical improvement [11]. However, limited data are available regarding the differential behavior of salivary MMPs following FMD, particularly in the context of psychosocial comorbidities such as depression.

The aim of this study was to assess short-term changes in salivary MMP-8 and MMP-9 following full-mouth disinfection and to explore their association with clinical periodontal parameters. Additionally, a subgroup analysis was performed to explore potential differences according to depressive comorbidity.

The null hypothesis was that no differences exist between salivary MMP-8 and MMP-9 in their responsiveness to full-mouth disinfection or in their association with clinical periodontal improvement and that depressive comorbidity does not significantly influence these outcomes.

## 2. Materials and Methods

### 2.1. Study Design

This prospective pre–post clinical study was designed to evaluate short-term changes in salivary MMP-8 and MMP-9 levels and clinical periodontal parameters following full-mouth disinfection (FMD) in patients with stage II periodontitis. Participants were allocated into two parallel groups according to the presence or absence of depressive comorbidity. The study was not designed as a controlled comparison between treatment modalities but as an observational assessment of biomarker and clinical changes after a standardized non-surgical periodontal intervention.

The study was performed in accordance with the ethical principles outlined in the Declaration of Helsinki for research involving human subjects. Ethical approval for the study protocol was obtained from the Research Ethics Committee of the “Grigore T. Popa” University of Medicine and Pharmacy, Iași, Romania (approval number 400/15 February 2024). All participants were informed about the purpose and procedures of the study prior to enrollment and provided written informed consent.

Participants were divided into two main study groups: patients diagnosed with periodontal disease without systemic conditions (PAR group) and patients presenting periodontal disease associated with a previously established depressive disorder (DEP group). Group allocation was based on pre-existing depressive comorbidity and was not randomized; therefore, comparisons between groups should be interpreted as exploratory.

Clinical periodontal examination and saliva collection were performed at two time points: at baseline, prior to the initiation of periodontal therapy, and after the completion of the Full-Mouth Disinfection (FMD) protocol.

Each group was further divided into two subgroups according to the moment of evaluation. The periodontal disease group included the subgroups PARi, representing the initial evaluation, and PARr, corresponding to the reevaluation after the FMD treatment. Similarly, the group of patients with periodontal disease associated with depression included the subgroups DEPi, representing the initial/baseline evaluation, and DEPr, corresponding to the post-treatment assessment. This classification allowed the comparison of biomarker levels and clinical periodontal parameters both between groups and between the two evaluation stages.

**Figure 1:**
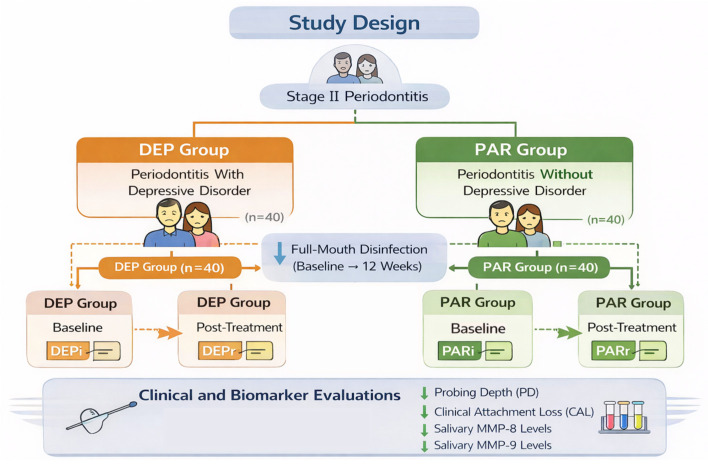
Study design.

The study stages are illustrated in Figure 1.

### 2.2. Sample Size Calculation

The required sample size for the present study was calculated using the G*Power software version 3.1. (Heinrich Heine University, Düsseldorf, Germany). The calculation was performed prior to participant recruitment in order to ensure adequate statistical power for detecting potential differences between the study groups.

An effect size of 0.4, corresponding to a large effect according to Cohen’s classification, was selected for the analysis. The significance level (α) was set at 0.05, while the statistical power (1 − β) was established at 0.80. Based on these parameters, the minimum required sample size was estimated to be 76 participants.

To account for potential dropouts and ensure sufficient statistical robustness, a total of 80 participants were ultimately included in the study and equally distributed between the two study groups.

### 2.3. Study Population

The study population consisted of 80 adult participants diagnosed with stage II periodontitis (moderate form), according to the 2017 World Workshop classification of periodontal and peri-implant diseases and conditions. Subjects were allocated into two groups based on the presence or absence of depressive disorder. The first group included patients with periodontitis without depressive comorbidity (PAR group), while the second group comprised patients diagnosed with periodontitis associated with depressive disorder (DEP group). Each group included 40 participants.

Participants were recruited from patients attending the Department of Periodontology, “Grigore T. Popa” University of Medicine and Pharmacy, Iași, Romania. For subjects included in the DEP group, the diagnosis of depressive disorder had been previously established by a qualified psychiatrist, independently of the study investigators, based on the Diagnostic and Statistical Manual of Mental Disorders, Fifth Edition (DSM-5) criteria. No standardized scale for assessing depression severity was applied; therefore, depressive disorder was treated as a categorical variable.

Eligible participants were aged between 25 and 65 years and presented with a confirmed clinical and radiological diagnosis of stage II periodontitis. Only patients with moderate disease severity were included to ensure homogeneity across study groups. Clinical periodontal evaluation included the assessment of probing depth (PD) and clinical attachment loss (CAL), which were used to characterize periodontal status at baseline and during follow-up.

Participants were evaluated at two time points: the initial examination (baseline) and a follow-up assessment performed 12 weeks after the application of the full-mouth disinfection (FMD) protocol. At both time points, saliva samples were collected for the determination of matrix metalloproteinase-8 (MMP-8) and matrix metalloproteinase-9 (MMP-9) levels, while PD and CAL were recorded to monitor changes in clinical periodontal status following therapy.

All participants received detailed information regarding the study protocol and provided written informed consent prior to inclusion. The study was conducted in accordance with ethical standards, and all subjects were cooperative throughout the study procedures.

### 2.4. Inclusion and Exclusion Criteria

Participants were selected according to predefined inclusion and exclusion criteria to ensure group homogeneity and to minimize potential confounding factors that could influence both clinical periodontal parameters and salivary biomarker levels.

*Inclusion Criteria*

Participants were eligible for inclusion if they met the following criteria:

- age between 25 and 65 years;
- confirmed clinical and radiological diagnosis of stage II periodontitis, according to the 2017 World Workshop classification;
- presence of Ramfjord teeth, allowing standardized periodontal assessment;
- ability to comply with the study protocol and attend follow-up visits.

For participants included in the DEP group, an additional inclusion criterion was a previously established diagnosis of depressive disorder, confirmed by a qualified psychiatrist based on the Diagnostic and Statistical Manual of Mental Disorders, Fifth Edition (DSM-5) criteria. Depression diagnosis was established prior to study inclusion and was independent of the study investigators.

*Exclusion Criteria*

Participants were excluded if they met any of the following criteria:

- presence of systemic diseases known to affect periodontal status (e.g., diabetes mellitus, autoimmune disorders);
- severe systemic diseases such as leukemia, cancer, or HIV infection;
- contraindications for ultrasonic periodontal instrumentation, including the presence of cardiac pacemakers or severe dentin hypersensitivity;
- pregnancy or lactation;
- ongoing anticoagulant therapy;
- use of antibiotics or anti-inflammatory medication within the previous six months;
- history of periodontal therapy within the last six months;
- smoking habit;
- absence of Ramfjord teeth;
- inability to comply with study procedures or follow-up visits.

### 2.5. Clinical Periodontal Examination

Prior to treatment, all participants underwent a comprehensive periodontal examination to confirm the diagnosis and establish baseline clinical parameters.

Periodontal measurements were performed using a CP15 periodontal probe (Carl Martin GmbH, Solingen, Germany). Probing depth (PD) and clinical attachment loss (CAL) were recorded at six sites per tooth for each Ramfjord tooth (1.6, 2.1, 2.4, 3.6, 4.1, and 4.4), including mesiobuccal, mid-buccal, distobuccal, mesiolingual/palatal, mid-lingual/palatal, and distolingual/palatal surfaces. The cementoenamel junction and gingival margin were used as anatomical reference points.

Clinical attachment loss was calculated by combining probing depth with the position of the gingival margin relative to the cementoenamel junction, providing an estimate of total periodontal attachment loss.

Examiner calibration was performed prior to the study to ensure measurement reproducibility. Two calibrated investigators independently assessed 10 subjects not included in the study sample. Inter-examiner agreement was evaluated using the intraclass correlation coefficient (ICC), demonstrating high reproducibility (ICC ≥ 0.85).

Due to the nature of the intervention, blinding of participants was not feasible.

### 2.6. Full-Mouth Disinfection (FMD) Protocol

All participants underwent non-surgical periodontal therapy based on the full-mouth disinfection (FMD) protocol. The treatment was performed in three consecutive phases: initial assessment and preparation, full-mouth disinfection procedure, and post-treatment reevaluation.

The FMD protocol was selected to ensure rapid and uniform reduction of the subgingival microbial load across all participants, thereby allowing a standardized assessment of biomarker dynamics following therapy.

*Initial Phase*

At baseline, clinical periodontal parameters, including probing depth (PD) and clinical attachment loss (CAL), were recorded. Saliva and gingival crevicular fluid samples were collected for the determination of matrix metalloproteinase-8 (MMP-8) and matrix metalloproteinase-9 (MMP-9) levels.

Dental plaque was disclosed to assess biofilm accumulation and to facilitate oral hygiene instruction. All participants received standardized oral hygiene guidance. Supragingival mechanical debridement was performed prior to subgingival instrumentation.

*Full-Mouth Disinfection Procedure*

The causal therapy phase consisted of comprehensive subgingival instrumentation performed according to the principles of the FMD protocol. Mechanical debridement was carried out using ultrasonic scalers followed by manual instrumentation, aiming to achieve thorough removal of subgingival biofilm and calculus.

Antiseptic irrigation was performed during the procedure to enhance microbial reduction. The full-mouth treatment was completed within a short time interval to minimize intraoral reinfection. The FMD treatment protocol is detailed in Table 1 [12].

Local supraperiosteal anesthesia was administered when required. Subgingival debridement was performed using ultrasonic scalers equipped with P3 tips, followed by manual instrumentation with Gracey Micro Mini Five curettes to ensure thorough removal of subgingival biofilm and calculus.

During instrumentation, periodontal sites were irrigated with antiseptic solutions containing 0.2% chlorhexidine and 3% hydrogen peroxide to enhance microbial reduction.

*Adjunctive Therapy*

Following completion of mechanical debridement, patients were instructed to rinse with 0.2% chlorhexidine solution two to three times daily for 14 days.

*Post-Treatment Reevaluation*

A follow-up examination was performed 12 weeks after completion of the FMD protocol. Clinical periodontal parameters, including probing depth (PD) and clinical attachment loss (CAL), were reassessed using the same methodology as at baseline. Oral hygiene instructions were reinforced, and additional instrumentation was performed when residual inflammation or biofilm accumulation was detected.

At the reevaluation visit, a second saliva sample was collected to assess changes in salivary matrix metalloproteinase-8 (MMP-8) and matrix metalloproteinase-9 (MMP-9) levels.

Full-mouth disinfection (FMD) represents a type of conventional scaling and root planing (SRP), in which subgingival debridement is performed within a short time frame, typically within 24 h, and is combined with adjunctive antiseptic measures to reduce bacterial load and prevent reinfection [11].

### 2.7. Saliva Collection

Unstimulated saliva samples were collected from all participants for the determination of salivary matrix metalloproteinase-8 (MMP-8) and matrix metalloproteinase-9 (MMP-9) levels under standardized conditions.

Participants were instructed to refrain from food intake, beverages (except water), and oral hygiene procedures for at least 30 min prior to sample collection. Before collection, subjects rinsed the oral cavity with water to remove debris and standardize the oral environment. Following the rinse, a short waiting period was observed to minimize potential dilution effects and to ensure that salivary biomarker levels were representative of the oral environment.

Unstimulated saliva was then collected over a standardized period of approximately 5 min, during which participants were instructed to passively accumulate saliva and expectorate into sterile disposable containers. Samples were transferred into sterile tubes using single-use devices. All samples were collected between 9:00 and 12:00 a.m. to minimize circadian variability.

Saliva samples were obtained at two time points: baseline (prior to periodontal therapy) and 12 weeks after completion of the full-mouth disinfection protocol. Following collection, samples were stored at 4 °C during transport and processed on the same day.

### 2.8. Biomarker Analysis

Salivary concentrations of matrix metalloproteinase-8 (MMP-8) and matrix metalloproteinase-9 (MMP-9) were determined using commercially available enzyme-linked immunosorbent assay (ELISA) kits, according to the manufacturers’ instructions.

MMP-8 levels were measured using the Human MMP-8 ELISA Kit (E-EL-H1450, Elabscience, Houston, TX, USA), while MMP-9 levels were determined using the Human MMP-9 ELISA Kit (E-EL-H6075, Elabscience, Houston, TX, USA). Both assays are based on quantitative sandwich ELISA methodology and are designed for the detection of human MMPs in biological samples.

Saliva samples were processed according to standardized laboratory procedures. All samples were analyzed in duplicate to ensure measurement reliability. Absorbance was measured using a microplate reader, and biomarker concentrations were calculated based on standard calibration curves generated for each assay.

Biomarker levels were assessed at two time points, baseline (prior to full-mouth disinfection) and 12 weeks after completion of the treatment protocol, in order to evaluate changes associated with periodontal therapy.

The assays were performed in accordance with the manufacturers’ protocols, and their sensitivity and detection ranges were consistent with the specifications provided.

### 2.9. Statistical Analysis

Statistical analyses were performed using IBM SPSS Statistics (version 29.0.0, IBM Corp., Armonk, NY, USA). Descriptive statistics were calculated for all variables and are presented as mean ± standard deviation (mean ± SD).

The primary analysis focused on evaluating changes over time and differences between groups for clinical periodontal parameters (probing depth [PD] and clinical attachment loss [CAL]) and salivary biomarkers (MMP-8 and MMP-9). To account for the repeated-measures design, a two-way mixed analysis of variance (ANOVA) was applied, with time (baseline vs. post-treatment) as the within-subject factor and group (periodontitis without depression vs. periodontitis with depressive comorbidity) as the between-subject factor. This approach allowed for the assessment of the main effects of time and group, as well as the interaction effect (time × group), which reflects potential differences in treatment response between groups.

When appropriate, post hoc comparisons were performed using Bonferroni correction to adjust for multiple testing. In addition to *p*-values, effect sizes were estimated using partial eta squared (η^2^p) for ANOVA analyses and Cohen’s d for pairwise comparisons in order to provide a measure of the magnitude of observed effects.

To further explore treatment-related changes, absolute differences (Δ values) between baseline and post-treatment measurements were calculated for all clinical and biomarker variables. These scores were used for between-group comparisons using independent samples *t*-tests.

The associations between salivary biomarker levels and clinical periodontal parameters were assessed using Pearson’s correlation coefficient. Correlation analyses were performed both on absolute values and on change scores to evaluate the relationship between biomarker dynamics and clinical improvement.

Assumptions of normality and homogeneity of variances were assessed using the Shapiro–Wilk test and Levene’s test, respectively. In cases where deviations from normality were observed, data distribution was further evaluated graphically, and results were interpreted with caution.

All statistical tests were two-tailed, and statistical significance was set at *p* < 0.05.

## 3. Results

The demographic characteristics of the study population are presented in Table 2. The PAR and DEP groups were comparable in terms of age and sex distribution, with no statistically significant differences observed between groups (*p* > 0.05). These findings indicate a balanced baseline profile, supporting the validity of subsequent comparisons between the study groups.

**Figure 2:**
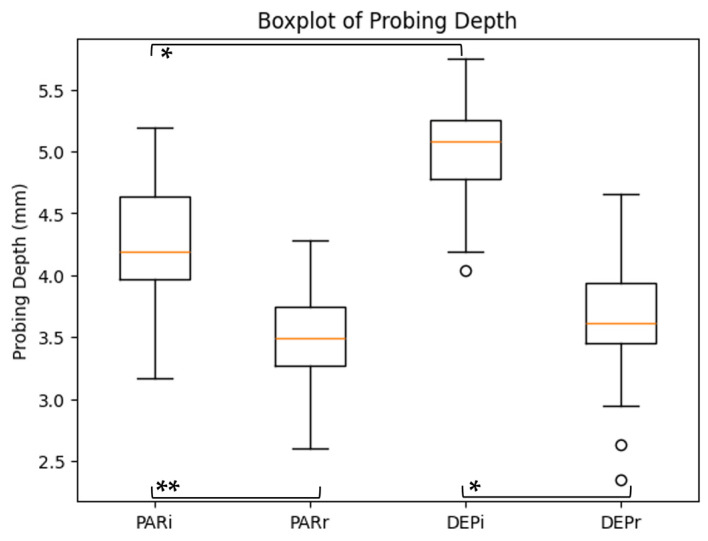
Distribution of probing depth across study subgroups. Statistical significance is indicated as follows: * *p* < 0.05; ** *p* < 0.01.

**Figure 3:**
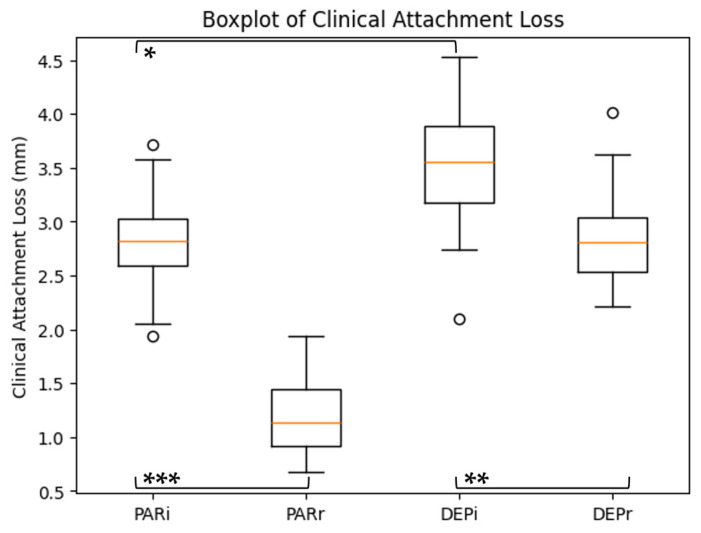
Distribution of clinical attachment loss across study subgroups. Statistical significance is indicated as follows: * *p* < 0.05; ** *p* < 0.01; *** *p* < 0.001.

The distribution of probing depth (PD) across study subgroups is illustrated in Figure 2, while the distribution of clinical attachment loss (CAL) is presented in Figure 3.

The mean salivary levels of MMP-8 and MMP-9 (mean ± SD, ng/mL) at baseline and post-treatment. are presented in Table 3.

A two-way mixed analysis of variance (ANOVA) was performed to evaluate the effects of time (baseline vs. post-treatment) and group (PAR vs. DEP) on clinical periodontal parameters.

For probing depth (PD), a significant main effect of time was observed (F = 62.4, *p* < 0.001, η^2^p = 0.45), indicating a reduction following periodontal therapy. The main effect of group was not statistically significant (F = 2.31, *p* = 0.132), suggesting comparable overall PD values between groups. The interaction effect (time × group) was not significant (F = 1.12, *p* = 0.293), indicating that the magnitude of PD reduction did not differ significantly between groups.

Similarly, for clinical attachment loss (CAL), a significant main effect of time was identified (F = 58.7, *p* < 0.001, η^2^p = 0.43), reflecting improvement after therapy. No significant main effect of group (F = 2.76, *p* = 0.101) or interaction effect (F = 1.34, *p* = 0.251) was observed.

Within-group comparisons (paired *t*-tests), summarized in Table 4, confirmed significant reductions in both PD and CAL in the PAR and DEP groups (PAR: PD *p* = 0.001, CAL *p* < 0.001; DEP: PD *p* = 0.003, CAL *p* = 0.002).

At baseline, inter-group differences were statistically significant (PD: *p* = 0.021; CAL: *p* = 0.018), while post-treatment differences were not significant (PD: *p* = 0.094; CAL: *p* = 0.072). The magnitude of change (Δ values) was comparable between groups.

### 3.1. Salivary Levels of MMP-8

Mean salivary MMP-8 levels at baseline and post-treatment are presented in Table 3.

A two-way mixed ANOVA revealed no statistically significant main effect of time for MMP-8 (F = 2.14, *p* = 0.148, η^2^p = 0.03), indicating the absence of significant short-term changes following therapy. The main effect of group (F = 1.87, *p* = 0.175) and the interaction effect (time × group) (F = 0.96, *p* = 0.331) were also not statistically significant.

Descriptive analysis showed minor decreases in MMP-8 levels (PAR: 1.4 ± 0.5 to 1.1 ± 0.4 ng/mL; DEP: 1.6 ± 0.6 to 1.3 ± 0.5 ng/mL), which did not reach statistical significance.

Within-group comparisons confirmed that changes in MMP-8 levels were not statistically significant in either group (PAR: *p* = 0.084; DEP: *p* = 0.097), as shown in Table 5.

### 3.2. Salivary Levels of MMP-9

A two-way mixed ANOVA demonstrated a significant main effect of time for MMP-9 (F = 35.6, *p* < 0.001, η^2^p = 0.31), indicating a reduction in salivary levels following periodontal therapy.

No statistically significant main effect of group (F = 1.54, *p* = 0.219) was observed. The interaction effect (time × group) was not statistically significant (F = 1.89, *p* = 0.173), indicating that the magnitude of change did not differ significantly between groups.

Descriptive analysis showed a marked decrease in MMP-9 levels (PAR: 2.4 ± 0.8 to 1.1 ± 0.5 ng/mL; DEP: 2.6 ± 0.9 to 1.5 ± 0.6 ng/mL).

Within-group comparisons confirmed significant reductions in both study groups (PAR: *p* = 0.002; DEP: *p* = 0.004), as presented in Table 5.

### 3.3. Correlation Analysis (Clinical Parameters and MMP Levels)

**Figure 4:**
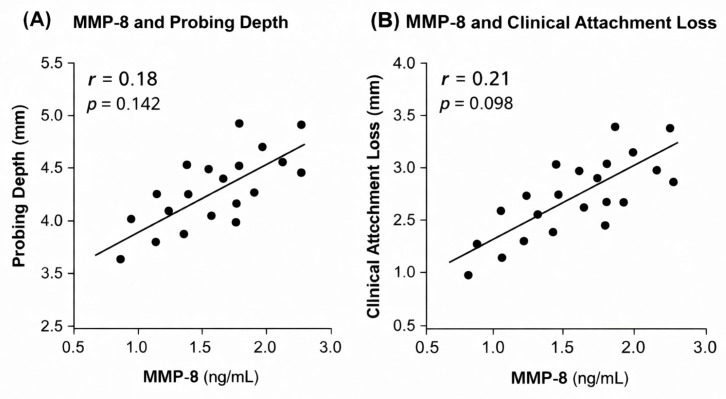
Correlation between salivary MMP-8 levels and clinical periodontal parameters.

**Figure 5:**
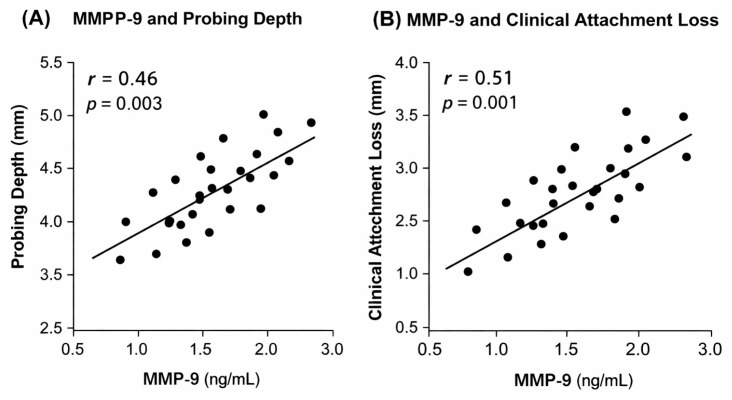
Correlation between salivary MMP-9 levels and clinical periodontal parameters.

The relationships between salivary MMP levels and clinical periodontal parameters are illustrated in Figure 4 and Figure 5.

Pearson correlation analysis indicated moderate positive relationships between salivary MMP-9 levels and clinical periodontal parameters, with statistically significant correlations observed for both probing depth (PD) and clinical attachment loss (CAL).

In contrast, correlations between salivary MMP-8 levels and clinical parameters were weak and did not reach statistical significance (PD: r = 0.18, *p* = 0.142; CAL: r = 0.21, *p* = 0.098).

### 3.4. ROC Curve Analysis

Receiver operating characteristic (ROC) curve analysis was performed to evaluate the ability of salivary biomarkers to discriminate between baseline and post-treatment status.

**Figure 6:**
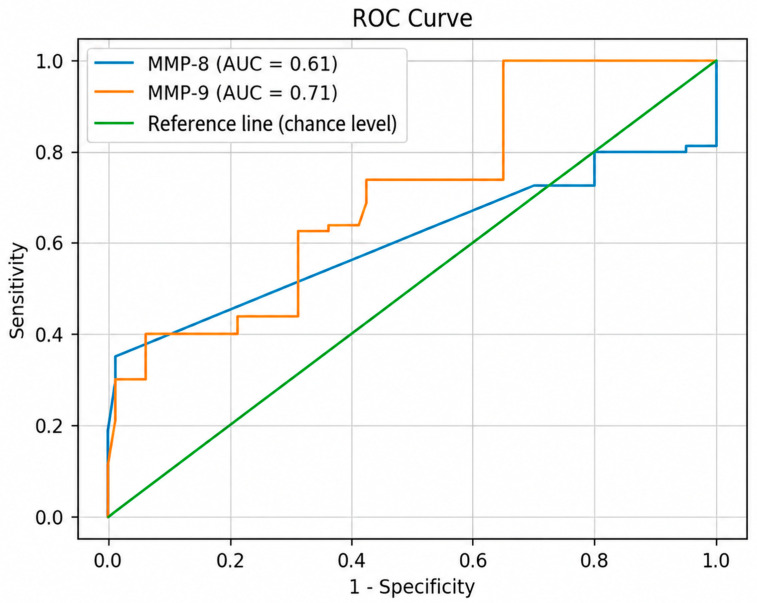
Receiver operating characteristic (ROC) curves for salivary MMP-8 and MMP-9 in distinguishing baseline from post-treatment status. MMP-9 demonstrated higher diagnostic performance compared to MMP-8.

ROC curve analysis (Figure 6) demonstrated that MMP-9 had a moderate diagnostic performance (AUC = 0.71), indicating acceptable discrimination between baseline and post-treatment status.

In contrast, MMP-8 showed limited discriminative ability (AUC = 0.61).

## 4. Discussion

The present study demonstrates that non-surgical periodontal therapy based on full-mouth disinfection (FMD) leads to significant clinical improvement, accompanied by a differential response of salivary biomarkers. Although both groups showed reductions in probing depth and clinical attachment loss, only MMP-9 exhibited a statistically significant decrease and moderate correlations with clinical parameters. In contrast, MMP-8 levels remained relatively unchanged and were not significantly associated with clinical outcomes, indicating that not all matrix metalloproteinases equally reflect short-term periodontal healing.

The divergent behavior of MMP-8 and MMP-9 can be interpreted in light of biomarker compartmentalization and molecular specificity. MMP-9 appears to be more closely related to acute inflammatory activity, as reflected by its consistent reduction following therapy. Conversely, the lack of significant variation in salivary MMP-8 may be attributable to the assessment of total MMP-8 in whole saliva, which may not adequately capture biologically active forms of the enzyme [13].

Although MMP-8 is a well-established marker of periodontal tissue breakdown, its diagnostic performance is strongly influenced by both the molecular form analyzed and the biological compartment [14]. Previous studies have shown that active MMP-8 (aMMP-8), particularly when measured in gingival crevicular fluid, provides a more accurate reflection of disease activity compared to total MMP-8 in saliva [15,16,17]. Therefore, the absence of significant changes observed in the present study likely reflects methodological and compartment-related limitations rather than a lack of biological relevance.

Taken together, these findings highlight that biomarker responsiveness is influenced not only by biological function but also by sampling strategy and analytical approach. While MMP-9 demonstrated higher sensitivity to short-term changes in inflammatory burden, salivary MMP-8 may require more specific detection methods or alternative sampling sites to reliably reflect periodontal dynamics [18].

These findings are consistent with previous studies reporting reductions in inflammatory and matrix-degrading biomarkers following non-surgical periodontal therapy, while also extending current knowledge by directly comparing the responsiveness of two extensively investigated MMPs within the same clinical setting [19,20]. The moderate correlations identified between MMP-9 and clinical periodontal parameters further support its potential role as a dynamic indicator of periodontal inflammation and therapeutic response. Moreover, the present results highlight the importance of biomarker selection when saliva is used as a diagnostic medium, as different molecules may exhibit variable sensitivity depending on both the biological compartment and the temporal stage of disease resolution.

From the perspective of full-mouth disinfection (FMD), the observed clinical improvements align with the established concept of rapid and comprehensive reduction of the subgingival microbial load, aimed at minimizing intraoral reinfection. Previous systematic reviews have generally demonstrated comparable clinical outcomes between FMD and conventional quadrant-based scaling and root planing, with variability largely influenced by treatment protocols and patient-related factors [21,22,23]. In this context, the magnitude of early clinical improvement observed in the present study supports the concept that intensive biofilm disruption may facilitate rapid periodontal stabilization.

A relevant methodological limitation is the absence of a parallel control group treated with conventional periodontal therapy. Consequently, the present study should not be interpreted as a comparative assessment of treatment modalities. Instead, FMD may be regarded as a standardized therapeutic model that enables the investigation of biomarker dynamics under conditions of rapid and uniform biofilm reduction, with the observed changes primarily reflecting the biological response to periodontal therapy [24,25,26].

The psychosocial dimension warrants careful interpretation. Patients with depressive disorder presented higher baseline periodontal parameters, suggesting a potential association with increased disease severity [4,27]. However, no significant differences in treatment response or biomarker dynamics were observed between groups following therapy. This apparent convergence may indicate that intensive non-surgical periodontal treatment is effective in reducing inflammation in the short term, regardless of underlying psychosocial conditions. Nevertheless, this finding should be interpreted with caution, as depression was assessed as a categorical variable without standardized evaluation of severity or information regarding ongoing treatment, which may have introduced heterogeneity within the study group. It is also possible that the impact of depression becomes more relevant during the maintenance phase rather than during active treatment [4,12,28].

Moreover, the inclusion of depressive comorbidity in this study should be interpreted as exploratory, as no significant differences were observed between groups. While psychosocial factors may influence periodontal disease, the present findings do not support a measurable short-term effect on clinical outcomes or biomarker dynamics under the conditions investigated.

From a clinical perspective, the present findings suggest that not all biomarkers are equally suitable for monitoring short-term periodontal response. The observed sensitivity of MMP-9 supports its potential utility as a non-invasive marker of treatment response, whereas MMP-8 may require alternative sampling strategies or more specific analytical approaches to demonstrate clinical relevance. The integration of salivary biomarkers into periodontal assessment could enhance patient monitoring and risk stratification, provided that methodological limitations are addressed and clinically meaningful thresholds are established [29,30].

Several limitations should be considered when interpreting these findings. First, the study design is based on pre- versus post-treatment comparisons without a parallel control group, limiting conclusions regarding comparative treatment effectiveness. Second, the follow-up period is relatively short and does not allow assessment of long-term stability or recurrence. Third, depression was treated as a categorical variable without a standardized severity assessment, which may have introduced heterogeneity within the DEP group. Also, salivary biomarkers are subject to pre-analytical variability, and MMP-8 in particular may require assessment of its active form or sampling closer to the lesion site to better reflect local disease activity. Moreover, the study evaluated a limited set of clinical periodontal parameters such as PD and CAL. Additional indices (the plaque index (PI) and the gingival bleeding index (GBI)) were not included and could have provided complementary information regarding oral hygiene status and inflammatory activity.

Another limitation of this study is the assessment of total MMP-8 in whole saliva, which may not accurately reflect its biologically active form or local periodontal conditions. The lack of significant changes observed for MMP-8 may therefore be related to methodological constraints rather than the absence of biological relevance.

Taken together, these limitations suggest that while the findings are robust for early treatment response, further longitudinal and methodologically refined studies are required.

## 5. Conclusions

Full-mouth disinfection (FMD) was associated with significant short-term improvements in clinical periodontal parameters, confirming its effectiveness as a non-surgical therapeutic approach.

Among the evaluated salivary biomarkers, MMP-9 demonstrated consistent reductions following therapy and moderate correlations with clinical parameters, supporting its potential utility as a non-invasive marker for monitoring short-term periodontal response.

In contrast, total salivary MMP-8 did not show significant short-term changes, suggesting limited sensitivity in this context when assessed in whole saliva. This finding highlights the importance of selecting appropriate biomarkers and analytical approaches in saliva-based diagnostics.

From a clinical perspective, salivary MMP-9 may represent a more reliable biomarker for evaluating early treatment response, whereas MMP-8 may require alternative sampling strategies or assessment of its active form to provide clinically relevant information.

No significant differences were observed between patients with and without depressive comorbidity; however, this result should be interpreted with caution due to the limited characterization of depressive status.

Further longitudinal and controlled studies are needed to define clinically applicable thresholds and to validate the role of salivary biomarkers in routine periodontal monitoring.

## Figures and Tables

**Table 1:** Overview of the Full-Mouth Disinfection (FMD) Treatment Protocol.

Stage	Clinical Procedures Performed
Initial evaluation	Recording periodontal parameters (PD, CAL); collection of saliva and gingival crevicular fluid; plaque disclosure; patient education and oral hygiene motivation; supragingival biofilm removal.
Full-mouth disinfection therapy	Administration of supraperiosteal local anesthesia when required; subgingival instrumentation performed using ultrasonic scalers (P3 tips) followed by manual scaling with Gracey Micro Mini Five curettes; antiseptic irrigation with 0.2% chlorhexidine and 3% hydrogen peroxide.
Adjunctive measures	Post-treatment rinsing with 0.2% chlorhexidine mouthwash 2–3 times daily for 14 days.
Follow-up assessment	Reevaluation at 12 weeks using the same clinical parameters (PD, CAL); reinforcement of oral hygiene instructions; additional instrumentation when residual periodontal inflammation is detected.

**Table 2:** Demographic characteristics of the study population.

Parameter	PAR (n = 40)	DEP (n = 40)	*p*-Value
Age (years, mean ± SD)	41.3 ± 8.7	43.9 ± 9.8	0.21
Sex (Male/Female), n	24/16	21/19	0.48

**Table 3:** Mean salivary levels of MMP-8 and MMP-9 (mean ± SD, ng/mL) at baseline and post-treatment, including intra-group comparisons (*p*-values).

Group	Subgroup	MMP-8 (ng/mL)	*p*-Values	MMP-9 (ng/mL)	*p*-Values
PAR	i (baseline)	1.4 ± 0.5	0.084	2.4 ± 0.8	0.002
PAR	r (post-treatment)	1.1 ± 0.4		1.1 ± 0.5	
DEP	i (baseline)	1.6 ± 0.6	0.097	2.6 ± 0.9	0.004
DEP	r (post-treatment)	1.3 ± 0.5		1.5 ± 0.6	

**Table 4:** Changes in clinical periodontal parameters within and between groups.

Parameter	Group	*p*-Value (Intra-Group)	*p*-Value (Inter-Group Baseline)	*p*-Value (Inter-Group Post)
PD (mm)	PAR	0.001	0.021	0.094
PD (mm)	DEP	0.003		
CAL (mm)	PAR	<0.001	0.018	0.072
CAL (mm)	DEP	0.002		

**Table 5:** Statistical analysis of salivary MMP-8 and MMP-9 levels (*p*-values).

Biomarker	Comparison	PAR (Intra-Group)	DEP (Intra-Group)	Inter-Group (Baseline)	Inter-Group (Post-Treatment)
MMP-8	Baseline vs. Post	0.084	0.097	0.412	0.276
MMP-9	Baseline vs. Post	0.002	0.004	0.318	0.067

## Data Availability

All data are contained within the article.

## Abbreviations

The following abbreviations are used in this manuscript:

Abbreviation	Explanation
AUC	Area under the curve
ANOVA	Analysis of variance
CAL	Clinical attachment loss
DSM-5	Diagnostic and Statistical Manual of Mental Disorders, Fifth Edition
ELISA	Enzyme-linked immunosorbent assay
FMD	Full-mouth disinfection
GBI	Gingival bleeding index
ICC	Intraclass correlation coefficient
MMP	Matrix metalloproteinase
MMP-8	Matrix metalloproteinase-8
MMP-9	Matrix metalloproteinase-9
aMMP-8	Active matrix metalloproteinase-8
PD	Probing depth
PI	Plaque index
SD	Standard deviation
SRP	Scaling and root planing
